# Updated Estimates of Neural Tube Defects Prevented by Mandatory Folic Acid Fortification — United States, 1995–2011

**Published:** 2015-01-16

**Authors:** Jennifer Williams, Cara T. Mai, Joe Mulinare, Jennifer Isenburg, Timothy J. Flood, Mary Ethen, Barbara Frohnert, Russell S. Kirby

**Affiliations:** 1 National Center on Birth Defects and Developmental Disabilities, CDC; 2 Carter Consulting; 3 Bureau of Public Health Statistics, Arizona Department of Health Services; 4 Birth Defects Epidemiology and Surveillance Branch, Texas Department of State Health Services; 5 Division of Community and Family Health, Minnesota Department of Health; 6 College of Public Health, University of South Florida

In 1992, the U.S. Public Health Service recommended that all women capable of becoming pregnant consume 400 *μ*g of folic acid daily to prevent neural tube defects (NTDs) (1). NTDs are major birth defects of the brain and spine that occur early in pregnancy as a result of improper closure of the embryonic neural tube, which can lead to death or varying degrees of disability. The two most common NTDs are anencephaly and spina bifida. Beginning in 1998, the United States mandated fortification of enriched cereal grain products with 140 *μ*g of folic acid per 100 g (2). Immediately after mandatory fortification, the birth prevalence of NTD cases declined. Fortification was estimated to avert approximately 1,000 NTD-affected pregnancies annually (2,3). To provide updated estimates of the birth prevalence of NTDs in the period after introduction of mandatory folic acid fortification (i.e., the post-fortification period), data from 19 population-based birth defects surveillance programs in the United States, covering the years 1999–2011, were examined. After the initial decrease, NTD birth prevalence during the post-fortification period has remained relatively stable. The number of births occurring annually without NTDs that would otherwise have been affected is approximately 1,326 (95% confidence interval = 1,122–1,531). Mandatory folic acid fortification remains an effective public health intervention. There remain opportunities for prevention among women with lower folic acid intakes, especially among Hispanic women, to further reduce the prevalence of NTDs in the United States.

In August 2014, a total of 19 population-based birth defects surveillance programs in the United States reported to CDC the number of cases of spina bifida (*International Classification of Diseases, 9th Revision, Clinical Modification* codes 741.0 and 741.9) and anencephaly (codes 740.0–740.1) among deliveries occurring during 1995–2011 among non-Hispanic whites, non-Hispanic blacks, and Hispanics, as well as all racial/ethnic groups combined. Surveillance programs were grouped by whether they systematically conducted prenatal ascertainment to capture diagnosed cases (eight sites: Arkansas, Georgia, Iowa, New York, Oklahoma, Puerto Rico, South Carolina, and Utah) or did not (11 sites: Arizona, California, Colorado, Illinois, Kentucky, Maryland, New Jersey, North Carolina, Texas, West Virginia, and Wisconsin). Programs with prenatal ascertainment monitored birth defects among live births, stillbirths, and elective terminations, and included collection of information from prenatal sources, such as prenatal diagnostic facilities.

The birth prevalences of spina bifida, anencephaly, and both NTDs combined were estimated as the total number of cases divided by the total number of live births during the pre-fortification (1995–1996) and post-fortification periods (1999–2011). These prevalence estimates were multiplied by the average number of live births in the United States for the selected periods to estimate the annual number of NTD cases nationwide. Prevalence estimates were also calculated by type of surveillance program (i.e., programs with prenatal ascertainment and programs without prenatal ascertainment) and maternal race/ethnicity (i.e., non-Hispanic white, non-Hispanic black, and Hispanic). The estimated annual number of NTDs prevented was calculated as the difference between the estimated annual number during the pre-fortification period and the estimated annual number during the post-fortification period using prevalence estimates from programs with prenatal ascertainment.

A decline in NTDs was observed for all three of the racial/ethnic groups examined between the pre-fortification and post-fortification periods (Figure). The post-fortification prevalence has remained relatively stable. During the observed periods, Hispanics consistently had a higher prevalence of NTDs compared with the other racial/ethnic groups, whereas non-Hispanic blacks generally had the lowest prevalence.

The birth prevalences of anencephaly and spina bifida during the pre-fortification (1995–1996) and post-fortification periods (biennial from 1999–2008, last 3 years of available data from 2009–2011, and all years from 1999–2011) for programs with and without prenatal ascertainment were estimated. Overall, a 28% reduction in prevalence was observed for anencephaly and spina bifida using data from all participating programs; a greater reduction (35%) was observed among programs with prenatal ascertainment than for programs without prenatal ascertainment (21%) (Table). The prevalence reported for anencephaly from programs with prenatal ascertainment was consistently higher across all racial/ethnic groups than for programs without prenatal ascertainment, whereas the difference in the observed prevalence of spina bifida was not as pronounced between the two types of programs. Based on data from programs that collect prenatal ascertainment information, an updated estimate of the number of births occurring annually without NTDs that would otherwise have been affected is 1,326 (95% confidence interval = 1,122–1,531).

## Discussion

The birth prevalence of NTDs during the post-fortification period has remained relatively stable since the initial reductions observed during 1999–2000, immediately after mandatory folic acid fortification in the United States. The updated estimate of approximately 1,300 NTD-affected births averted annually during the post-fortification period is slightly higher than the previously published estimate (3). Factors that could have helped contribute to the difference include a gradual increase in the number of annual live births in the United States during the post-fortification period and data variations caused by differences in surveillance methodology. The lifetime direct costs for a child with spina bifida are estimated at $560,000, and for anencephaly (a uniformly fatal condition), the estimate is $5,415 (4); multiplying these costs by the NTD case estimates translates to an annual saving in total direct costs of approximately $508 million for the NTD-affected births that were prevented.

The reduction in NTD cases during the post-fortification period inversely mirrors the increase in serum and red blood cell (RBC) folate concentrations among women of childbearing age in the general population. Fortification led to a decrease in the prevalence of serum folate deficiency from 30% to <1%, and a decrease in the prevalence of RBC folate deficiency from 6% to no measureable deficiency (5). A recent study modeled the dose-response relationship between RBC folate concentrations in women of childbearing age and risk for NTDs. It showed that RBC folate concentrations >1,000 nmol/L were sufficient to substantially attenuate the risk for NTDs at a population level (6). Using data from the National Health and Nutrition Examination Survey for 1988–2010 (5) and adjusting for assay differences, the estimated mean RBC folate concentration in women aged 15–44 years in the United States is 1,290–1,314 nmol/L, which appears to indicate that for many women of childbearing age, current strategies are preventing a majority of folic acid–sensitive NTDs (5,6). However, almost a quarter (21.6%) of women of childbearing age in the United States still do not have RBC folate concentrations associated with a lower risk for NTDs, and targeted strategies might be needed to achieve RBC folate concentrations >1,000 nmol/L in this group (7).

Although a reduction in the birth prevalence of NTDs has been observed for all three of the racial/ethnic groups examined, the prevalence among Hispanics is consistently greater than that among other racial/ethnic groups. Possible reasons could include differences in folic acid consumption and genetic factors affecting the metabolism of folic acid. Fewer Hispanic women (17%) than non-Hispanic white women (30%) report consuming ≥400 *μ*g of folic acid per day through fortified food or supplements (8). A common genetic polymorphism in Hispanics, the methylenetetrahydrofolate reductase T allele, has been associated with relatively lower plasma folate and RBC folate concentrations compared with those without this polymorphism (9). Persons with this polymorphism have more genetic susceptibility to a folate insufficiency. To target Hispanics who might need additional folic acid intake to prevent NTDs, one strategy under consideration in the United States is to fortify corn masa flour with folic acid at the same level as enriched cereal grain products. Implementation of corn masa flour fortification would likely prevent an additional 40 cases of NTDs annually (10).

What is already known on this topic?

A decline in the prevalence of neural tube defects (NTDs) was reported during the period immediately after mandatory folic acid fortification in the United States, which translated to approximately 1,000 births occurring annually without anencephaly or spina bifida that would otherwise have been affected.

What is added by this report?

The prevalence of NTDs during the post-fortification period has remained relatively stable since the initial reduction observed immediately after mandatory folic acid fortification in the United States. Using the observed prevalence estimates of NTDs during 1999–2011, an updated estimate of the number of births occurring annually without NTDs that would otherwise have been affected is 1,300.

What are the implications for public health practice?

Current fortification efforts should be maintained to prevent folic acid–sensitive NTDs from occurring. There are still opportunities for prevention among women with lower folic acid intakes, especially among Hispanic women, to further reduce the prevalence of NTDs in the United States.

The findings in this report are subject to at least one limitation. The prevalence data used in this study might not be generalizable to the entire United States, but only to the extent that NTD prevalence in other states/territories not examined could differ from NTD prevalence in the states/territories represented in this analysis.

The initial decline in NTD prevalence reported immediately after mandatory folic acid fortification has been maintained after more than a decade since implementation. Mandatory folic acid fortification remains an effective public health policy intervention.

## Figures and Tables

**FIGURE:**
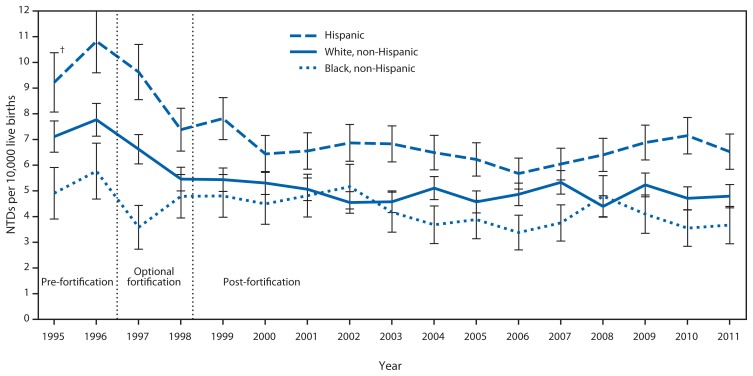
Prevalence of neural tube defects (NTDs) (anencephaly and spina bifida) before and after mandatory folic acid fortification, by maternal race/ethnicity — 19 population-based birth defects surveillance programs,* United States, 1995–2011 * Contributing programs are based in Arkansas, Arizona, California, Colorado, Georgia, Illinois, Iowa, Kentucky, Maryland, New Jersey, New York, North Carolina, Oklahoma, Puerto Rico, South Carolina, Texas, Utah, West Virginia, and Wisconsin. ^†^ 95% confidence interval.

**TABLE:** Prevalence (per 10,000 live births) and estimated average annual number of spina bifida and anencephaly cases, by period and prenatal ascertainment status — 19 population-based birth defects surveillance programs, United States, 1995–1996 and 1999–2011*

Type of case/Prenatal ascertainment status	Pre-fortification	Post-fortification							Difference in estimated annual cases between pre- and post-fortification
	
	1995–1996	1999–2000	2001–1002	2003–2004	2005–2006	2007–2008	2009–2011	1999–2011	
**Anencephaly**									
Programs with prenatal ascertainment†									
Prevalence	4.2	3.3	3.2	2.7	2.9	2.9	2.8	2.9	
Estimated annual cases (95% CI)	1,628 (1,440 –1,816)	1,305 (1,139–1,471)	1,277 (1,113–1441)	1,105 (950–1,260)	1,222 (1,059–1,384)	1,231 (1,067–1,394)	1,127 (1,000–1,255)	1,206 (1,142–1,269)	422 (298–547)
Programs without prenatal ascertainment§									
Prevalence	2.3	2.3	1.9	1.9	1.7	1.8	1.9	1.9	
Estimated annual cases (95% CI)	913 (827–1,000)	924 (847–1,001)	774 (704–845)	778 (708–849)	701 (634–768)	763 (693–833)	760 (703–817)	781 (754–809)	
**Spina bifida**									
Programs with prenatal ascertainment†									
Prevalence	6.5	4.0	4.5	3.7	4.0	4.4	3.7	4.0	
Estimated annual cases (95% CI)	2,549 (2,314–2,785)	1,617 (1,433–1,802)	1,792 (1,598–1,986)	1,517 (1,336–1,698)	1,678 (1,487–1,868)	1,869 (1,668–2,070)	1,476 (1,330–1,622)	1,645 (1,571–1,719)	904 (743–1,066)
Programs without prenatal ascertainment§									
Prevalence	4.3	3.5	3.3	3.5	3.2	3.4	3.6	3.4	
Estimated annual cases (95% CI)	1,685 (1,568–1,803)	1,405 (1,310–1,501)	1,326 (1,234–1,418)	1,426 (1,330–1,521)	1,328 (1,236–1,420)	1,455 (1,359–1,551)	1,443 (1,365–1,521)	1,401 (1,364–1,438)	
**Anencephaly and spina bifida**									
Programs with prenatal ascertainment†									
Prevalence	10.7	7.3	7.6	6.4	6.9	7.2	6.5	7.0	
Estimated annual cases (95% CI)	4,177 (3,876–4,479)	2,922 (2,674–3,170)	3,069 (2,815–3,323)	2,622 (2,384–2,860)	2,899 (2,649–3,150)	3,100 (2,840–3,359)	2,604 (2,410–2,797)	2,851 (2,754–2,948)	1,326 (1,122–1,531)
Programs without prenatal ascertainment§									
Prevalence	6.7	5.8	5.2	5.4	4.8	5.2	5.5	5.3	
Estimated annual cases (95% CI)	2,599 (2,453–2,745)	2,329 (2,206–2,452)	2,100 (1,984–2,216)	2,204 (2,085–2,322)	2,029 (1,915–2,143)	2,218 (2,100–2,337)	2,203 (2,107–2,299)	2,182 (2,136–2,228)	
**Average annual live births** ¶	3,895,542	4,009,116	4,023,830	4,101,001	4,201,952	4,281,964	4,027,880	4,101,490	

**Abbreviation:** CI = confidence interval.

* Estimated annual number of cases for the specified period is calculated by multiplying the prevalence by the average number of U.S. annual live births. Data during the optional fortification period (1997–1998) are not presented.

† States with prenatal ascertainment (n = 8): Arkansas, Georgia, Iowa, New York, Oklahoma, Puerto Rico, South Carolina, and Utah.

§ States without prenatal ascertainment (n = 11): Arizona, California, Colorado, Illinois, Kentucky, Maryland, New Jersey, North Carolina, Texas, West Virginia, and Wisconsin.

¶ Data available at http://wonder.cdc.gov.
